# Effects of Gait and Cognitive Task Difficulty on Cognitive-Motor Interference in Aging

**DOI:** 10.1155/2012/583894

**Published:** 2012-11-07

**Authors:** Prudence Plummer-D'Amato, Briana Brancato, Mallory Dantowitz, Stephanie Birken, Christina Bonke, Erin Furey

**Affiliations:** Department of Physical Therapy, Northeastern University, 360 Huntington Avenue, 6 Robinson Hall, Boston, MA 02115-5000, USA

## Abstract

Although gait-related dual-task interference in aging is well established, the effect of gait and cognitive task difficulty on dual-task interference is poorly understood. The purpose of this study was to examine the effect of gait and cognitive task difficulty on cognitive-motor interference in aging. Fifteen older adults (72.1 years, SD 5.2) and 20 young adults (21.7 years, SD 1.6) performed three walking tasks of varying difficulty (self-selected speed, fast speed, and fast speed with obstacle crossing) under single- and dual-task conditions. The cognitive tasks were the auditory Stroop task and the clock task. There was a significant Group × Gait Task × Cognitive Task interaction for the dual-task effect on gait speed. After adjusting for education, there were no significant effects of gait or cognitive task difficulty on the dual-task effects on cognitive task performance. The results of this study provide evidence that gait task difficulty influences dual-task effects on gait speed, especially in older adults. Moreover, the effects of gait task difficulty on dual-task interference appear to be influenced by the difficulty of the cognitive task. Education is an important factor influencing cognitive-motor interference effects on cognition, but not gait.

## 1. Introduction

Functional community ambulation requires an ability to perform cognitive tasks while walking and an ability to adapt to extrinsic environmental factors that increase the complexity of mobility, such as obstacle avoidance (e.g., curbs) and time-critical tasks (e.g., crossing the street within the time constraints imposed by traffic signals) [1]. A reduced capacity for dual-task walking may limit community mobility. Research has shown that healthy older adults experience significant decrements in gait speed when cognitive tasks are performed while walking [2], a phenomenon referred to as dual-task interference or cognitive-motor interference. 

A limitation of the existing research on dual-task interference in aging is that it has focused predominantly on dual-task performance during unobstructed walking at preferred gait speed. Therefore, the effect of dual-task interference on gait and cognitive performance during more attention-demanding gait tasks remains largely unknown. Furthermore, because slowing down during unobstructed walking in the gait laboratory is inconsequential for successful completion of the motor task, individuals may place more importance on performing the cognitive task [3]. Indeed, healthy young and older adults appear to place greater priority on the secondary (nongait) task in many dual-task situations [4, 5]. It seems reasonable to assume that when gait task complexity increases and there is a greater potential threat to stability, individuals would place more importance on completing the gait task safely. The current study explores how gait task difficulty affects cognitive-motor interference.

Bock et al. [4] examined the dual-task costs of performing cognitive and gait tasks of varying difficulty in both young and older adults. The authors concluded that in dual-task conditions, older adults were at greater risk for falls than young adults. However, the effect of task difficulty on dual-task interference was not explicitly analyzed and remains unclear. Numerous studies have examined dual-task effects on gait during obstacle avoidance in older adults [6–15], but none have directly compared the dual-task effects during obstacle negotiation to those during unobstructed walking. Kelly et al. [16] recently examined the effects of walking task difficulty (usual walking versus narrow-based walking) on dual-task performance in young adults. They found that walking task difficulty affected walking performance but not cognitive performance. Because this study included only dual-task conditions with specific instructions to focus primarily on either the cognitive task or the gait task, it is not known what effect task difficulty has on a person's default prioritization; that is, the way in which the person chooses to allocate his attention in the absence of explicit instruction. Moreover, the study included only young adults, so age-related differences in the effects of task difficulty on dual-task interference are not currently known.

The purpose of this study was to determine the effect of gait and cognitive task difficulty on dual-task interference in healthy young adults and older adults. We specifically compare obstructed and unobstructed gait because of the relevance to community ambulation. We hypothesized that as the difficulty of the gait task increased, attention to gait would increase, resulting in smaller dual-task effects relative to simple walking (unobstructed at self-selected speed). We focus on gait speed as the measure of gait performance in dual-task conditions, since meta-analysis results show that cognitive-motor interference effects across a range of cognitive tasks are prominent in gait speed [2]. Furthermore, because research has shown that dual-task effects on gait vary according to the type of cognitive task [17], we explore our hypothesis in two different cognitive-motor dual-task combinations. 

## 2. Methods

### 2.1. Participants

Twenty young adults (mean age 21.7 years, range 20–27) and 15 older adults (mean age 72.1 years, range 66–84) were recruited through advertisements at Northeastern University and local senior centers. To be included, participants had to be 18–30 years or older than 65 years, be able to walk independently in the community for at least 50 meters, have intact cognition according to the Mini-Mental State Exam (score > 23), and have normal or corrected-to-normal hearing and vision. Participants were excluded if they had a history of neurological disorders, any orthopedic conditions affecting gait, reported more than 2 falls in the past year, had an acute hospital stay within the last 3 months, or had a lower extremity amputation. Participants who met the selection criteria signed an informed consent form. The study was approved by the Institutional Review Board at Northeastern University. 

Demographic information was collected for each participant, including age, gender, and education. Cognitive abilities of the participants were characterized using the Mini-Mental State Exam [18], Digit Substitution Test [19], Stroop Color-Word Interference Test [20], Comprehensive Trail Making Tests [21], and the Activities-specific Balance Confidence (ABC) Scale [22]. Functional mobility was assessed using the Timed Up and Go test (TUG) [23]. 

### 2.2. Procedures

Each participant performed three gait tasks and two different cognitive tasks in isolation (single-task conditions) and each gait task in combination with each of the cognitive tasks (dual-task conditions). The cognitive tasks were the auditory Stroop [24] and the “clock task” [25]. In the auditory Stroop task, participants heard the words “high” and “low” spoken in either a high pitch (360 Hz) or a low pitch (180 Hz). The participants were instructed to indicate the pitch of the word they heard (ignoring the actual word presented) by responding verbally “high” or “low” as accurately and as quickly as possible. In the clock task, the participants heard a time (e.g., one-twenty-five) and were required to determine whether the two hands of the clock at the given time were in the same half (left/right) or opposite halves. If the hands were the same half, participants were asked to respond “yes;” if the hands were in opposite halves, the participants were asked to respond “no.” There were no clock-task stimuli in which one of the hands was exactly on the twelve or six (e.g., one-thirty). For each task, the participants completed at least two practice blocks of 30 trials while sitting. Single-task performance in each cognitive task was recorded in sitting and was always performed immediately before the dual-task conditions. Both the Stroop and clock tasks were produced using DirectRT (Empirisoft, New York, NY, USA). The stimuli were delivered through wireless headphones and participant responses were recorded through a wireless microphone (Logitech, Newark, CA). For both cognitive tasks, we measured reaction time (in milliseconds) and accuracy (percent of correct responses). Our assumption was that the clock task was more difficult because it required greater cognitive processing.

The three gait tasks were walking at self-selected speed (SS), walking at fastest comfortable speed (FC), and walking at fastest comfortable speed while stepping over an obstacle (OB). The three gait tasks were chosen to provide differing levels of task difficulty. A critical assumption was that walking at fastest comfortable speed was more attention-demanding than walking at preferred (self-selected) speed, and that walking fast and stepping over an obstacle further increased the demands of the task. All gait tasks involved participants walking across a 6.1-meter Platinum GAITRite walkway, which contains pressure activated sensors. The associated software computes spatio-temporal parameters of gait. The participants started and finished each pass 2 meters beyond the end of the walkway so that only steady state gait data were captured. Participants completed 6 passes of the walkway for each condition and the average of the 6 passes was used for analysis. In the OB condition, a 15 cm high hurdle was placed at the 4.5 m mark of the GAITRite walkway. The order of the three gait tasks was randomized, but was performed in the same order for the single-task and the two dual-task (Stroop, clock) blocks. Block order (single, Stroop, clock) was also randomized across participants. 

### 2.3. Measures of Dual-Task Interference

To analyze the effect of task difficulty on dual-task interference, we calculated the dual-task effect (DTE) on both gait speed and cognitive task performance (reaction time and accuracy). Dual-task effects on gait speed (DTEg) and clock and Stroop-task accuracy (DTEacc) were calculated as follows [16]:

(1) $$\begin{array}{c} \text{DTE}=\frac{\left(\text{dual}\;\;\text{task}-\text{single}\;\;\text{task}\right)}{\text{single}\;\;\text{task}}\times 100\%. \end{array}$$

Whereas a decrease in gait speed and accuracy represent performance decrement, an increase in reaction time (i.e., slower response) represents performance decline, therefore DTE on reaction time (DTErt) was calculated as follows [16]:

(2) $$\begin{array}{c} \text{DTE}=\frac{-\left(\text{dual}\;\;\text{task}-\text{single}\;\;\text{task}\right)}{\text{single}\;\;\text{task}}\times 100\%. \end{array}$$

Thus, for each variable, negative values for DTE indicate that performance deteriorated under dual-task conditions (i.e., dual-task cost), and positive values represent an improvement in the dual-task condition relative to single-task performance (i.e., dual-task benefit). 

### 2.4. Statistical Analysis

The young and older adults were compared on profile measures using independent *t*-tests. To verify whether the clock task required greater cognitive processing than the Stroop task, we conducted paired *t*-tests on mean reaction times for the two tasks in the single-task (sitting) condition for each group. To determine whether participants increased their SS gait speed as instructed in the FC and OB conditions, we analyzed changes in gait speed across conditions with a 3 Gait Task (SS, FC, OB) × 3 Cognitive Task (single, Stroop, clock) ANOVA for each group. Tukey's post hoc tests were used as needed. 

To analyze the effects of gait and cognitive task difficulty on dual-task interference, we applied a 3 Gait Task (SS, FC, OB) × 2 Cognitive Task (Stroop, clock) × 2 Group (young, older) repeated measures ANCOVA with education (years) as covariate to each dependent variable (DTEg, DTErt, DTEacc). Significant three-way interactions were followed up with two-way analyses and post hoc tests as needed. The partial eta squared (*η*_*p*_^2^) is presented as a measure of effect size for each repeated measures ANCOVA. By convention, 0.01 indicates a small effect size, 0.06 is moderate, and 0.14 represents a large effect [26]. Due to technical issues three older adults and one young adult were missing gait data from one or more of the gait tasks. Listwise deletion meant that these subjects were excluded from the analyses for gait variables, resulting in minor variations in degrees of freedom. All analyses were performed using SPSS 18.0 (SPSS Inc., Chicago, IL, USA).

## 3. Results

The young and older adults did not differ in global cognition assessed using the MMSE, but there were significant differences in specific cognitive domains, including executive function, inhibition of habitual response, and speed of processing (Table 1). The older adults also had significantly lower balance self-efficacy and took longer to complete the TUG. However, the differences between the groups on the cognitive and mobility measures were not considered clinically meaningful, since the older adults performed within normal limits for their age [21, 27, 28]. On average, the young adults had more years of education than the older adults (Table 1).

The mean reaction time while sitting (single-task) for the clock task (young adults *M* = 1403 ms, SD = 271; older adults *M* = 1889 ms, SD = 433) was significantly longer than that for the Stroop task (young adults *M* = 805 ms, SD = 109; older adults *M* = 956 ms, SD = 216) for both young adults, *t*(19) = −10.5, *P* < .001, *d* = 2.9, and older adults, *t*(14) = −9.5, *P* < .001, *d* = 2.7. Thus, consistent with our assumption, the clock task required greater cognitive processing than the Stroop task. In both tasks, mean reaction time of older adults was slower than young adults. 

### 3.1. Task Difficulty and Dual-Task Interference on Gait Speed

The mean gait speeds for young and older adults in each condition and each gait task are shown in Table 2. Participants significantly increased their walking speed when instructed to walk at their fastest comfortable speed. Among young adults, there were no significant differences in average gait speed between FC and OB for single-task or dual-task walking (Table 2). However, for older adults, average gait speed in OB was significantly slower than FC (Table 2). Table 2 also illustrates that there were significant declines in gait speed during the Stroop and clock tasks relative to single-task walking in both groups during the obstacle condition. Both groups had a significant reduction in FC walking speed for the clock task but not the Stroop task. In the SS condition, the older adults reduced their gait speed during Stroop but not clock task. Young adults had no significant change in gait speed in either dual-task in SS.

**Figure 1:**
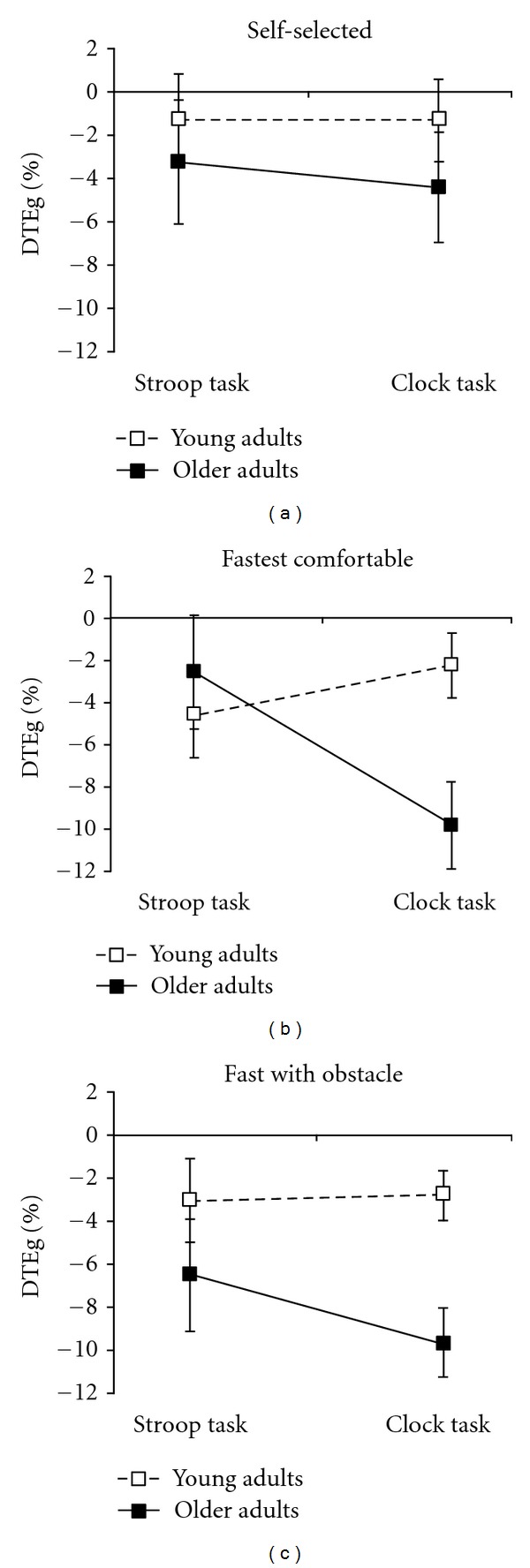
Dual-task effects on gait speed (DTEg) shown as a function of cognitive task and group for (a) walking at self-selected speed, (b) walking at fastest comfortable speed, and (c) walking at fastest comfortable speed and stepping over on obstacle. The interaction between cognitive task and group was significant for the fast comfortable walking condition (b), but not for the other two gait tasks. Error bars indicate standard error of the mean. DTEg are adjusted values for education in the Gait Task × Cognitive Task × Group ANCOVA; *n* = 19 young adults, *n* = 12 older adults.

These findings are corroborated by the significant Gait Task × Cognitive Task × Group interaction effect on DTEg, *F*(2,56) = 3.17, *P* = .050, *η*_*p*_^2^ = .10. Although the statistical significance was marginal, the effect size was large. Follow up two-way ANCOVAs revealed that the three-way interaction occurred because the Cognitive Task × Group interaction was significant for the FC gait task (*P* < .001; *η*_*p*_^2^ = .39), but not for SS (*P* = .794; *η*_*p*_^2^ < .01) or OB (*P* = .376; *η*_*p*_^2^ = .03) (Figure 1). Specifically, in FC, the dual-task cost (negative DTE) on gait speed for the older adults during the clock task was significantly greater than that of young adults, and was also greater than DTEg during the Stroop task for either group (Figure 1). In SS and OB, there was no effect of cognitive task on DTEg for either group, although Figure 1 shows a tendency for a larger dual-task cost in the clock task in OB among older adults.

### 3.2. Task Difficulty and Dual-Task Interference on Cognition

Young adults had mean accuracy of 99% (SD 0.01) in the Stroop task and 97.2% (SD 0.1) in the clock task. Older adults, on average, were significantly less accurate than young adults (*P* < .001), with lower mean accuracy in the clock task (*M* = 83.6%, SD = 0.2) than the Stroop task (*M* = 93.1%, SD = 0.1) (*P* = .045). Mean values for DTErt and DTEacc for each gait task and cognitive task are presented in Table 3. After adjusting for education, there were no significant main effects or interaction effects in the Gait Task × Cognitive Task × Group ANCOVA on DTErt or DTEacc. As illustrated by the wide confidence intervals in Table 3, there was large variability in the dual-task effects on both reaction time and accuracy for the Stroop and clock tasks.

Education was significantly related to DTEacc in the OB condition (*r* = .38, *P* = .026); lower levels of education were weakly associated with larger dual-task costs in accuracy on the clock task during obstacle crossing. Before adjusting for education, the three-way interaction on DTEacc was significant due to a Gait Task × Cognitive Task interaction for older adults but not young adults: dual-task cost on accuracy was significantly greater in the clock task during OB than in any other task among older adults; the effect was not significant after adjusting for education. 

## 4. Discussion

The purpose of this study was to determine the effect of gait and cognitive task difficulty on dual-task interference in aging. An important finding was that young adults were able to maintain fast walking speed in the obstructed condition with or without a simultaneous cognitive task, whereas older adults could not maintain fast walking speed in the obstructed condition even when no additional cognitive task was required. However, fast-obstacle walking speeds for older adults were still significantly faster than self-selected gait speed during single-task and Stroop task, but not in the clock task. In other words, although older adults reduced their gait speed in the fast-obstructed condition relative to fast-unobstructed walking, they were still able to walk faster than their preferred speed, except when they had to perform the clock task while stepping over the obstacle. This suggests that among older adults the attentional demands of performing a difficult cognitive task interfere with the attention processing requirements of negotiating an obstacle. Indeed, obstacle negotiation requires attention to spatial characteristics of gait in order to adjust strides and avoid hitting the obstacle. It is likely that the older adults slowed down as an adaptive safety strategy to avoid making motor errors when stepping over the obstacle, and that this effect was exaggerated when the added cognitive task demanded greater attentional resources. 

Consistent with previous research [17], older adults demonstrated a significant dual-task decline in gait speed during the Stroop task whereas young adults did not. Young adults, however, experienced a significant dual-task decline in gait speed during the Stroop task in the fast-obstacle condition. This finding suggests that in more attention-demanding gait tasks such as obstacle avoidance, a relatively simple cognitive task can impact gait speed, even in healthy young adults. Whereas the Stroop task affected walking speed in the fast-obstructed condition but not the fast-unobstructed condition, the clock task significantly reduced gait speed in both fast-obstructed and fast-unobstructed walking conditions. Thus, more difficult cognitive tasks may amplify dual-task interference in gait speed in easier gait tasks. The three-way interaction on DTEg corroborates the findings for gait speed and provides evidence for differential effects of gait and cognitive task difficulty on cognitive-motor interference during walking between young and older adults. 

We hypothesized that increasing the attentional demands of gait would reduce the dual-task costs on gait speed due to increased allocation of attentional resources required for the gait task. In contrast to our hypothesis, there was a tendency for dual-task effects on gait speed to increase with increasing gait task difficulty, although this was only significant for the older adults in the clock task in the FC condition (see Figure 1). It is possible that because we did not instruct the participants where to prioritize their attention during the dual-task conditions, they chose to slow down to optimize safety and/or to maintain performance on the cognitive task. The large variability in the dual-task effects however, especially for cognitive task performance, implies that participants used a range of strategies to perform the dual-tasks. Future research should concentrate on identifying whether personal characteristics influence how individuals spontaneously allocate their attention during gait-related dual-task situations and whether particular subgroups of older adults are vulnerable to the effects of task difficulty.

An important finding from this study was the effect that controlling for education had on the dual-task interference effects on cognition. Analysis of the unadjusted means showed that the dual-task cost on accuracy in the clock task was significantly greater during obstacle avoidance than in any of the other gait conditions, but only for older adults. However, after controlling for between-group differences in education, the Cognitive Task by Gait Task interaction for older adults was no longer significant. The lack of significant interaction effects for cognitive variables after controlling for education suggests that education may play an important role in counteracting dual-task costs on cognitive task performance, especially accuracy. It may be that more education leads to fewer errors in cognitive processing during dual-task walking, regardless of the difficulty of the gait or cognitive task. That is, greater education may increase cognitive reserve and thereby reduce susceptibility to dual-task interference. The idea that education contributes to cognitive reserve, and that increased cognitive reserve can limit clinical expression of cognitive changes is supported by strong evidence from the field of dementia research [29, 30].

Although we tried to simulate the challenges of real-world walking demands by adding elements of speed and obstacle negotiation to our gait tasks, a limitation of this study is that the research was still conducted in a quiet research laboratory. Thus, it remains unknown how real-world environmental factors (e.g., noise, distraction) affect dual-task interference. Furthermore, we assumed a hierarchical increase in gait task difficulty between walking at self-selected speed, walking at fastest comfortable speed, and walking fast while stepping over an obstacle. However, we did not ask the participants of their perceptions of the tasks. Finally, the findings from this study may be limited in their generalizability due to the small sample size and the predominance of women in the sample. Thus, the findings should be viewed as preliminary; investigations involving larger, more representative samples are needed to examine the interactions between age group, gait task difficulty, and cognitive task difficulty on cognitive-motor interference. In the future, manipulating the timing of the onset of the stimulus in the obstacle negotiation path may provide more insight into the interactions between attentional processing associated with obstacle avoidance and the attention processing of an additional cognitive task.

## 5. Conclusions

In conclusion, the results of this study suggest that obstacle negotiation at fast walking speed, such as when stepping up a curb to avoid traffic, is highly attention-demanding for older adults and significantly compromises the ability to maintain walking speed. This study provides evidence that gait task difficulty influences dual-task effects on gait speed, especially in older adults. Moreover, the effects of gait task difficulty on dual-task interference appear to be influenced by the difficulty of the cognitive task. Education and/or cognitive reserve may be an important factor influencing cognitive-motor interference, especially in terms of performance of the cognitive task.

## Figures and Tables

**Table 1:** Characteristics (mean, SD) of sample.

Variable	Young adults (*n* = 20)	Older adults (*n* = 15)	*P* ^†^
Age	21.7 (1.6)	72.1 (5.2)	<.001
Female gender (number, %)	18 (90%)	12 (80%)	.418
Education (years)	15.3 (0.7)	12.4 (2.0)	<.001
MMSE^a^ (max. 30)	28.6 (1.1)	27.5 (2.2)	.080
Activities-specific balance confidence scale (max. 100)	95.8 (4.9)	85.9 (10.9)	.004
Digit symbol copy (time in seconds to complete)	57.4 (6.9)	84.1 (21.0)	.001
Digit symbol substitution (number correct in 90 seconds)	74.2 (9.1)	46.1 (13.2)	<.001
Stroop color-word interference^b^	24.1 (7.5)	35.8 (13.0)	.005
Comprehensive trail-making test interference^c^ (seconds)	13.8 (9.7)	35.1 (24.8)	.006
TUG (seconds)	7.4 (0.7)	9.6 (2.4)	.003

^†^ *t*-test for independent samples.

Abbreviations: MMSE: Mini Mental State Examination; TUG: Timed Up and Go test; *P* value is for *t*-test comparing young and older adults.

^a^MMSE measures global cognitive function; Digit symbol modalities test measures speed of processing and attention; Stroop and Trail Making Tests measure executive function; TUG measures balance during functional performance.

^b^Stroop interference score calculated as difference in number correct between baseline condition and interference condition.

^c^Trail-making test interference score calculated as difference in time (seconds) between time to complete Trail 5 and Trail 1.

**Table 2:** Mean (SD) gait speeds (m/s) for each condition and each gait task. Values are only from the subjects with gait speed data for all conditions.

Gait task	Young adults (*n* = 19)								Older adults (*n* = 12)							
	Single-task		Dual-stroop		Dual-clock		Mean		Single-task		Dual-stroop		Dual-clock		Mean	
SS	1.50	(0.17)	1.49	(0.16)	1.46	(0.22)	**1.48**	(0.17)	1.31	(0.29)	1.24^a^	(0.26)	1.28	(0.31)	**1.28**	(0.29)
FC	1.97^b^	(0.21)	1.90	(0.17)	1.90^a,b^	(0.20)	**1.93** ^ b^	(0.19)	1.51^b^	(0.37)	1.44^b^	(0.39)	1.39^a,b^	(0.36)	**1.44** ^ b^	(0.37)
OB	1.96^c^	(0.21)	1.88^a,c^	(0.19)	1.87^a,c^	(0.21)	**1.90** ^ c^	(0.20)	1.42^c,d^	(0.40)	1.32^a,c,d^	(0.36)	1.30^a,d^	(0.36)	**1.35** ^ d^	(0.37)
Mean	**1.81**	(0.17)	**1.76** ^ a^	(0.15)	**1.75** ^ a^	(0.18)	**1.77**	(0.24)	**1.41**	(0.35)	**1.33**	(0.33)	**1.32** ^ a^	(0.34)	1.36*	(0.24)

SS: walking at self-selected speed, FC: walking at fastest comfortable speed, OB: walking at fastest comfortable speed with obstacle crossing.

^a^Significant differences between single-task and dual-task (none of the differences between the two dual-tasks were significant), *P* ≤ .05 (Tukey's HSD).

^b^Significant differences between self-selected and fast, *P* ≤ .05 (Tukey's HSD).

^c^Significant differences between self-selected and fast obstacle, *P* ≤ .05 (Tukey's HSD).

^d^Significant differences between fast and fast obstacle, *P* ≤ .05 (Tukey's HSD).

*Significant difference between older adults and young adults, *P* < .001.

**Table 3:** Adjusted means (95% confidence intervals) for dual-task effects on reaction time (DTErt) and accuracy (DTEacc) for each cognitive task as a function of gait task. Positive values indicate a dual-task benefit relative to single-task; negative values indicate a dual-task cost relative to single-task performance. None of the main effects or interactions in the ANCOVA were significant for DTErt or DTEacc.

	DTErt (%)			DTEacc (%)		
	Self-selected	Fast comfortable	Fast obstacle	Self-selected	Fast comfortable	Fast obstacle
Stroop						
Young	−7.6 (−14.8, −0.4)	−0.5 (−8.1, 7.2)	−1.0 (−8.7, 6.6)	−0.1 (−6.0, 5.7)	−0.7 (−5.9, 4.6)	−0.5 (−5.4, 4.4)
Older	−3.7 (−12.4, 5.0)	−5.2 (−14.5, 4.0)	−4.2 (−13.4, 5.1)	−0.4 (−7.4, 6.7)	−1.2 (−7.5, 5.2)	0.2 (−5.6, 6.1)
Clock						
Young	4.7 (−3.0, 12.5)	18.4 (11.2, 25.7)	11.3 (3.8, 18.7)	−1.6 (−9.7, 6.6)	0.4 (−10.7, 11.5)	−1.8 (−9.4, 5.8)
Older	14.5 (5.1, 23.9)	14.7 (5.9, 23.5)	12.9 (3.8, 21.9)	−4.6 (−14.4, 5.3)	−2.4 (−15.9, 11.0)	−11.3 (−20.6, −2.1)
